# Microbiota analysis optimization for human bronchoalveolar lavage fluid

**DOI:** 10.1186/s40168-019-0755-x

**Published:** 2019-10-29

**Authors:** Pierre H. H. Schneeberger, Janice Prescod, Liran Levy, David Hwang, Tereza Martinu, Bryan Coburn

**Affiliations:** 1 Departments of Medicine and Laboratory Medicine & Pathobiology, University of Toronto, Toronto, M5G 1L7 Canada; 2 Department of Medicine, Division of Infectious Diseases, University Health Network, Toronto, Canada

**Keywords:** Lung microbiota, Sequencing accuracy, Sequencing precision, bronchoalveolar lavage

## Abstract

**Background:**

It is now possible to comprehensively characterize the microbiota of the lungs using culture-independent, sequencing-based assays. Several sample types have been used to investigate the lung microbiota, each presenting specific challenges for preparation and analysis of microbial communities. Bronchoalveolar lavage fluid (BALF) enables the identification of microbiota specific to the lower lung but commonly has low bacterial density, increasing the risk of false-positive signal from contaminating DNA. The objectives of this study were to investigate the extent of contamination across a range of sample densities representative of BALF and identify features of contaminants that facilitate their removal from sequence data and aid in the interpretation of BALF sample 16S sequencing data.

**Results:**

Using three mock communities across a range of densities ranging from 8E+ 02 to 8E+ 09 16S copies/ml, we assessed taxonomic accuracy and precision by 16S rRNA gene sequencing and the proportion of reads arising from contaminants. Sequencing accuracy, precision, and the relative abundance of mock community members decreased with sample input density, with a significant drop-off below 8E+ 05 16S copies/ml. Contaminant OTUs were commonly inversely correlated with sample input density or not reproduced between technical replicates. Removal of taxa with these features or physical concentration of samples prior to sequencing improved both sequencing accuracy and precision for samples between 8E+ 04 and 8E+ 06 16S copies/ml. For the lowest densities, below 8E+ 03 16S copies/ml BALF, accuracy and precision could not be significantly improved using these approaches. Using clinical BALF samples across a large density range, we observed that OTUs with features of contaminants identified in mock communities were also evident in low-density BALF samples.

**Conclusion:**

Relative abundance data and community composition generated by 16S sequencing of BALF samples across the range of density commonly observed in this sample type should be interpreted in the context of input sample density and may be improved by simple pre- and post-sequencing steps for densities above 8E+ 04 16S copies/ml.

## Background

The human microbiome is composed of organ-specific microbiota, the composition and function of which have been associated with a broad array of human diseases [1–5]. Complex microbial communities with specific exposure/disease associations have been identified in the lungs, even in individuals where the lung was previously considered to be sterile [6–10]. Importantly, much of what we understand of the microbiota in lung diseases is derived from populations with relatively high bacterial burden, such as those with infections or suppurative lung diseases including cystic fibrosis and bronchiectasis. The total biomass observed in samples from individuals with suppurative lung diseases is generally high, with low bacterial diversity and domination by a single taxon in a significant proportion of individuals [11, 12]. Less extensively studied samples collected from individuals with non-suppurative lung diseases can have much lower bacterial biomass but higher relative diversity [13].

Samples used to infer or directly measure the composition of the lung microbiota include oropharyngeal swabs or washes, sputum samples, bronchial aspirates, bronchoalveolar lavage fluids (BALF), and endobronchial biopsies [13]. The microbial community of sputum samples and bronchial aspirates is commonly contaminated with bacterial taxa present in the oral cavity [14, 15]; as such, these may not be the optimal sample types to study microbiome-disease interactions of the lower respiratory tract. BALF and bronchial mucosa biopsy samples usually present bacterial density 2–4 logs lower than the upper airway but harbour bacterial communities which are specific to the lower respiratory tract [13, 16].

Analysis of the bacterial communities from BALF samples is challenging, especially due to the low biomass commonly observed in these samples, making them more susceptible to artefacts introduced during sample processing and sequencing [17–19]. The relative contribution of contaminating taxa to BALF microbiota across the range of bacterial densities has not been systematically addressed, despite significant potential implications for the analysis of lung microbiota in a range of diseases.

Using bacterial communities with defined composition (‘mock communities’) across the range of bacterial densities observed in BALF, we quantified the accuracy and precision of 16S rRNA gene sequencing for the characterization of bacterial communities, characterized the features of contaminants and mock community taxa, analysed the impact of simple pre- and post-sequencing techniques on these performance characteristics, and developed post-sequencing filtering approaches based on our observations. Our goal was to assess the performance of 16S rRNA gene sequencing across the range of densities observed in human BALF samples to calibrate the interpretation of observational studies from human cohorts.

## Results

### Sequencing accuracy and precision over a range of input bacterial densities

#### Density range of BALF samples and mock communities

**Fig. 1:**
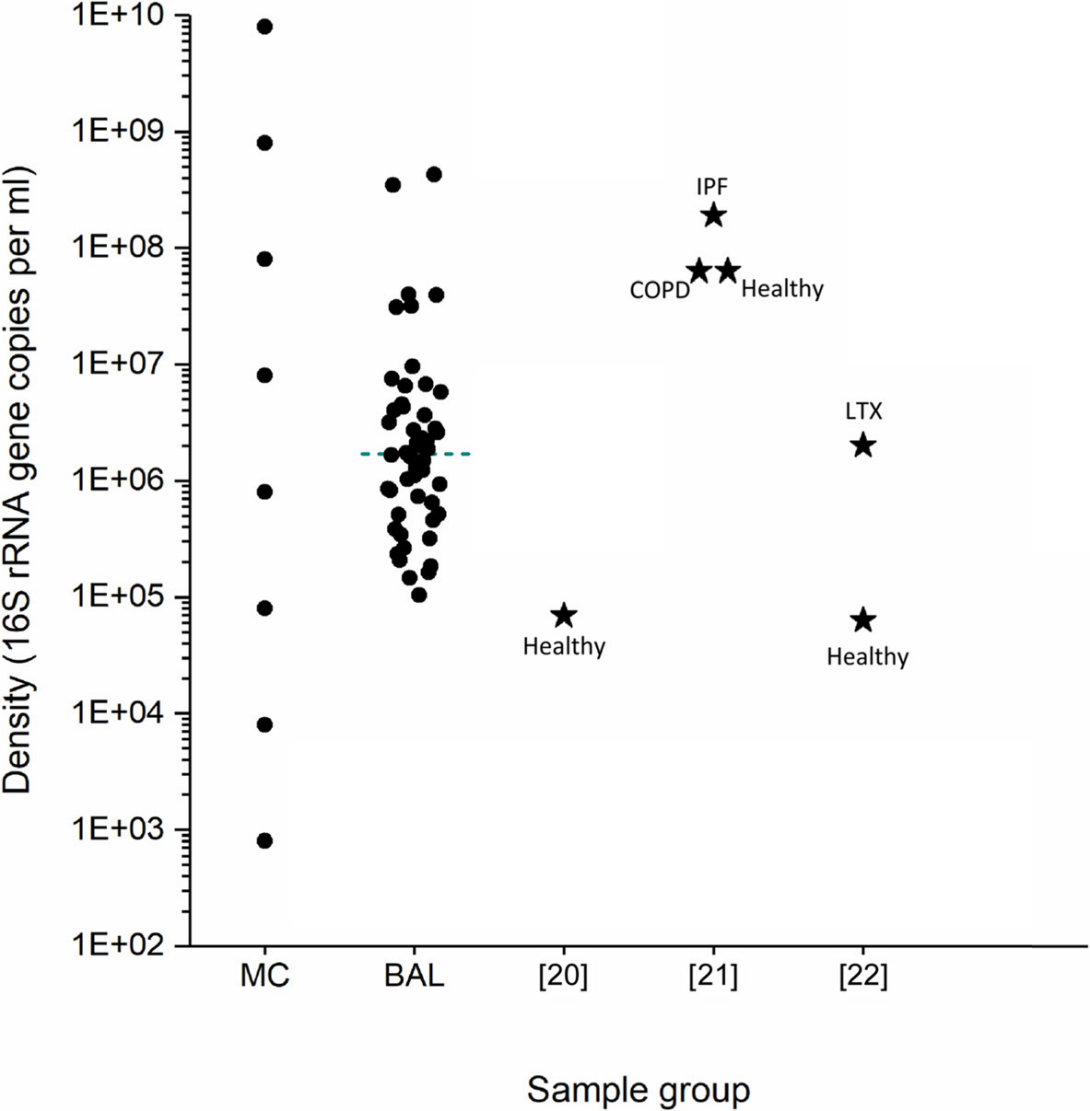
Comparison of bacterial load in BALF samples from patients with different conditions and mock communities tested in this study. 16S rRNA gene density in BALF samples and cultured mock communities. BALF, bronchoalveolar lavages fluids; rRNA, ribosomal ribonucleic acid; COPD, chronic obstructive pulmonary disease; IPF, idiopathic pulmonary fibrosis; LTX, lung transplant. Population mean for each reference (in brackets) is indicated with a star. MC = mock communities; BAL = study subset of BAL samples; [20] = Healthy; [21] = Healthy/COPD/IPF; [22] = Healthy/LTX

In order to calibrate the input densities of our mock communities, we measured the densities of a set of BALF samples obtained from the Toronto Lung Transplant Program (TLTP) Biobank by 16S qPCR (Fig. 1**)**. BALF samples ranged from 1E+ 05 to 4.2E+ 08 16S rRNA copies per millilitre (16S copies/ml, median 1.68E+ 06 16S copies/ml) and mock communities ~ 10^3^–10^10^ 16S copies/ml.

#### Composition and alpha diversity measure over density range

**Fig. 2:**
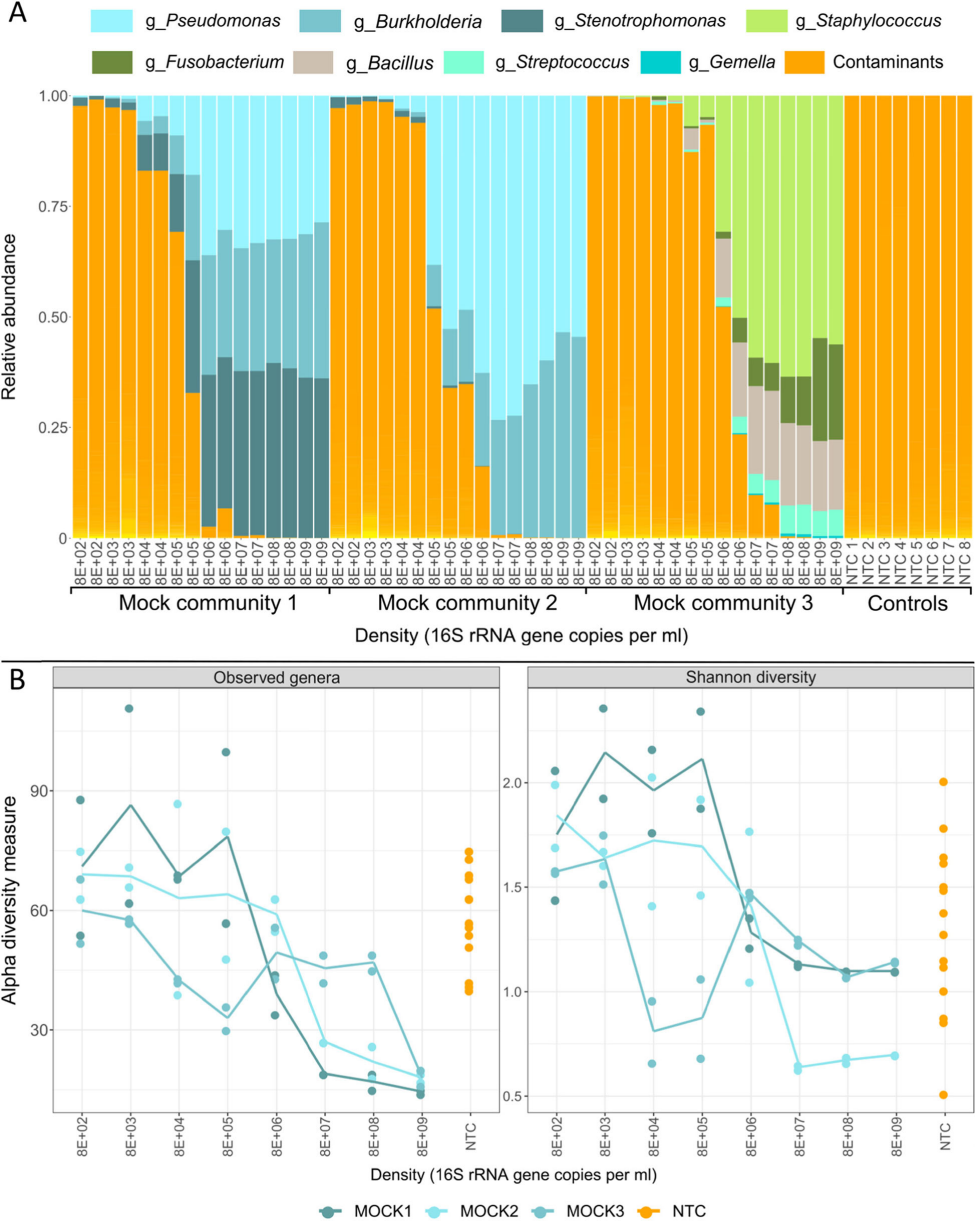
Taxonomic composition and alpha diversity indices of mock communities. **a** Histograms displaying the relative abundance of bacterial species from the mock community members versus contaminants over a range of input bacterial density. **b** Alpha diversity indices measured for mock communities at each input bacterial density. The solid line indicates the average value, for each input density. ml = millilitre

The proportion of reads assigned to genera within the input community increased with increasing sample density until an input bacterial density of ~ 8E+ 06 16S copies/ml for community 1 and 8E+ 07 16S copies/ml for communities 2 and 3 (Fig. 2a). Below an input density of 8E+ 04 (mock 1 and 2) and 8E+ 05 (mock 3) 16S copies/ml, the majority of reads were of non-mock community members (contaminants).

Below an input bacterial density of 8E+ 07 16S copies/ml, the number of observed genera was inversely correlated with input bacterial density (Fig. 2b). At densities < 8E+ 06 16S copies/ml, the number of observed genera approximated the number observed in no-template sequencing controls.

**Fig. 3:**
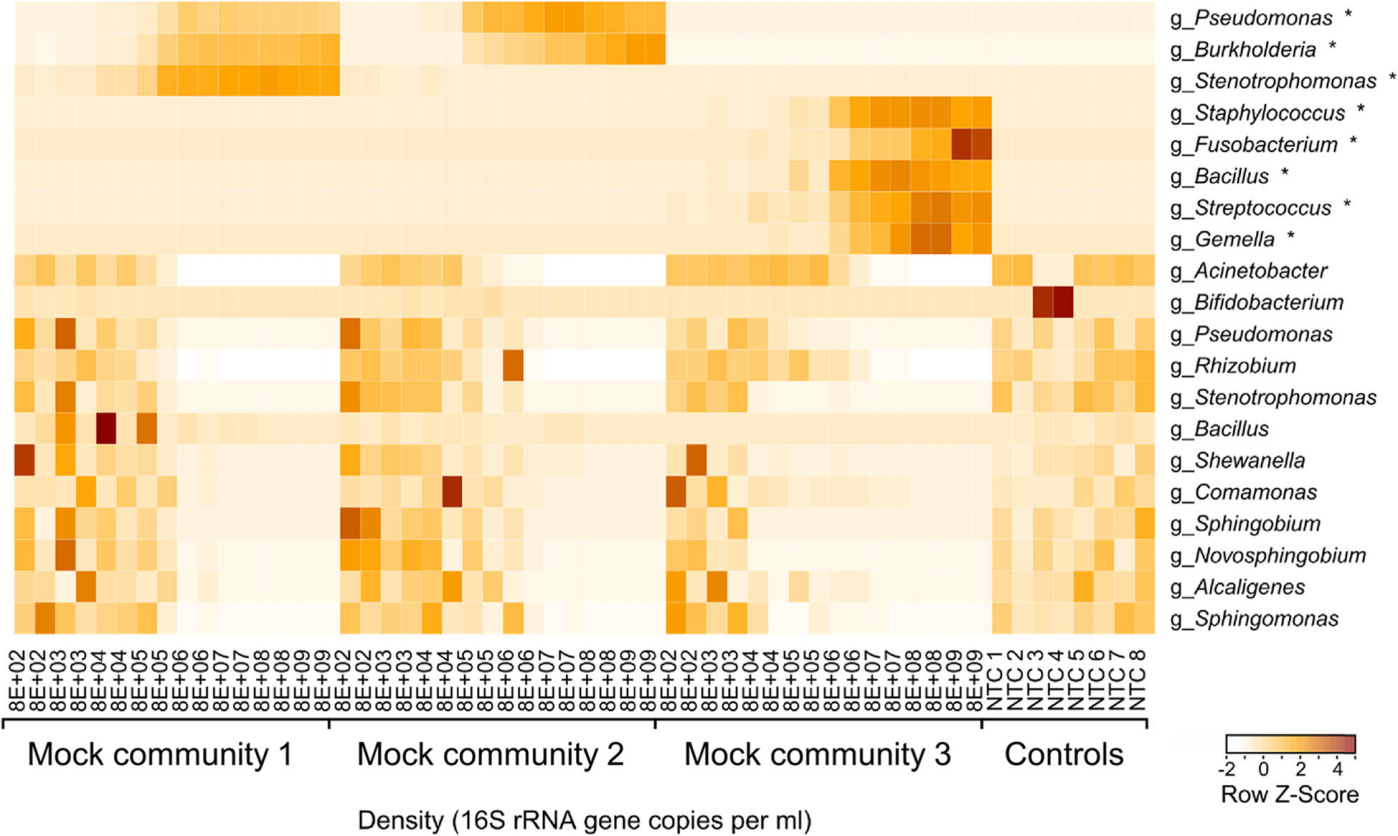
Heatmap showing the *Z*-scores of mock community species (*n* = 8) and the 12 most prevalent contaminating taxa across the complete dataset. Mock community species are highlighted with an asterisk (*)

The relative abundance *z*-score of the 20 most abundant taxa across the range of input densities is shown in Fig. 3. The most abundant genera in no-template controls were *Acinetobacter* and *Bifidobacterium*. *Acinetobacter*, *Pseudomonas* (not identified as *aeruginosa*), and *Rhizobium* were the most abundant contaminating taxa in mock community samples with an input density below 8E+ 05 16S copies/ml for mock community 1 and 8E+ 06 16S copies/ml for mock communities 2 and 3. These three genera cumulatively represented 81.3% of the contaminating taxa in the mock samples across the whole dataset. We assessed the relationship between relative abundance of each operational taxonomic unit (OTU) and sample input density (measured with 16S qPCR) using a Spearman correlation and summarized the 45 most abundant OTUs in all 3 communities in Table 1. Taxa which were members of the mock community were positively correlated with sample density (0.81 < *r*_s_ < 0.98), whereas contaminants were negatively correlated (− 0.98 < *r*_s_ < − 0.67), in agreement with prior studies [23]. Notably, OTUs which were mock community members but also commonly contaminants (e.g. *S*. *maltophilia*) were positively correlated in samples in which they were mock community members, and negatively correlated in communities where they were contaminants.

**Table 1:** Spearman correlation analysis to identify relations between relative abundances of bacterial taxa and sample input densities measured with qPCR

	r_s_ (all)	*P* (all)	r_s_ (M1)	*P* (M1)	r_s_ (M2)	*P* (M2)	r_s_ (M3)	*P* (M3)
Mock community members OTUs								
OTU: s_*Pseudomonas aeruginosa*	0.84	0.0001	0.81	0.0002	0.83	0.0001	− 0.43	0.1014
OTU: g_*Burkholderia*	0.99	0.0001	0.97	0.0001	0.98	0.0001	− 0.41	0.1146
OTU: g_*Staphylococcus*	0.91	0.0001	− 0.94	0.0001	− 0.62	0.0122	0.91	0.0001
OTU: s_*Stenotrophomonas maltophilia*	0.91	0.0001	0.92	0.0001	− 0.93	0.0001	− 0.89	0.0001
OTU: g_*Bacillus*	0.79	0.0005	− 0.67	0.0053	− 0.37	0.1627	0.85	0.0001
OTU: g_*Fusobacterium*	0.94	0.0001	0.06	0.8380	N.A	N.A	0.94	0.0001
OTU: s_*Streptococcus anginosus* subsp. *whileyi*	0.93	0.0001	− 0.28	0.2895	N.A	N.A	0.93	0.0001
OTU: s_*Gemella haemolysans*	0.75	0.0014	− 0.55	0.0305	− 0.26	0.3351	0.81	0.0002
Contaminants OTUs								
OTU: g_*Acinetobacter*	− 0.93	0.0001	− 0.96	0.0001	− 0.92	0.0001	− 0.79	0.0005
OTU: g_*Pseudomonas*	− 0.97	0.0001	− 0.92	0.0001	− 0.93	0.0001	− 0.97	0.0001
OTU: f_*Comamonadaceae*	− 0.93	0.0001	− 0.97	0.0001	− 0.88	0.0001	− 0.86	0.0001
OTU: s_*Acinetobacter indicus*	− 0.85	0.0001	− 0.85	0.0001	− 0.87	0.0001	− 0.81	0.0002
OTU: f_*Enterobacteriaceae*	− 0.97	0.0001	− 0.84	0.0001	− 0.96	0.0001	− 0.65	0.0082
OTU: g_*Rhizobium*	− 0.79	0.0004	− 0.94	0.0001	− 0.81	0.0002	− 0.86	0.0001
OTU: s_*Shewanella xiamenensis*	− 0.98	0.0001	− 0.85	0.0001	− 0.93	0.0001	− 0.67	0.0062
OTU: g_*Comamonas*	− 0.91	0.0001	− 0.89	0.0001	− 0.81	0.0002	− 0.89	0.0001
OTU: o_*Bacillales*	0.33	0.2098	− 0.25	0.3521	− 0.32	0.2259	0.90	0.0001
OTU: g_*Novosphingobium*	− 0.94	0.0001	− 0.81	0.0002	− 0.91	0.0001	− 0.49	0.0546
OTU: g_*Sphingobium*	− 0.98	0.0001	− 0.84	0.0001	− 0.94	0.0001	− 0.40	0.1216
OTU: s_*Alcaligenes faecalis* subsp. *parafaecalis*	− 0.96	0.0001	− 0.84	0.0001	− 0.85	0.0001	− 0.82	0.0002
OTU: g_*Paracoccus*	− 0.83	0.0001	− 0.84	0.0001	− 0.73	0.0019	− 0.48	0.0647
OTU: s_*Stenotrophomonas rhizophila*	− 0.95	0.0001	− 0.83	0.0001	− 0.95	0.0001	− 0.76	0.0011
OTU: g_*Bifidobacterium*	− 0.82	0.0001	− 0.86	0.0001	− 0.71	0.0028	− 0.18	0.5035
OTU: f_*Rhodobacteraceae*	− 0.90	0.0001	− 0.92	0.0001	− 0.89	0.0001	− 0.76	0.0010
OTU: g_*Stenotrophomonas*	− 0.95	0.0001	− 0.84	0.0001	− 0.91	0.0001	− 0.75	0.0013
OTU: s_*Pseudomonas beteli*	− 0.93	0.0001	− 0.83	0.0001	− 0.89	0.0001	− 0.63	0.0107
OTU: s_*Brevundimonas mediterranea*	− 0.92	0.0001	− 0.90	0.0001	− 0.81	0.0002	− 0.81	0.0002
OTU: s_*Pseudomonas psychrotolerans*	− 0.93	0.0001	− 0.76	0.0009	− 0.90	0.0001	− 0.68	0.0052
OTU: s_*Rhizobium cellulosilyticum*	− 0.92	0.0001	− 0.80	0.0003	− 0.92	0.0001	− 0.41	0.1153
OTU: g_*Sphingomonas*	− 0.97	0.0001	− 0.85	0.0001	− 0.82	0.0002	− 0.60	0.0163
OTU: g_*Brevundimonas*	− 0.94	0.0001	− 0.88	0.0001	− 0.83	0.0001	− 0.79	0.0004
OTU: s_*Sphingomonas aestuarii*	− 0.90	0.0001	− 0.82	0.0001	− 0.74	0.0016	− 0.71	0.0028
OTU: s_*Pseudomonas peli*	− 0.87	0.0001	− 0.83	0.0001	− 0.93	0.0001	− 0.81	0.0002
OTU: o_*Lactobacillales*	0.85	0.0001	− 0.36	0.1693	− 0.17	0.5305	0.85	0.0001
OTU: g_*Dietzia*	− 0.89	0.0001	− 0.76	0.0011	− 0.75	0.0012	− 0.52	0.0428
OTU: s_*Enhydrobacter aerosaccus*	− 0.45	0.0815	− 0.40	0.1216	− 0.27	0.3155	0.25	0.3516
OTU: f_*Staphylococcaceae*	0.47	0.0652	− 0.24	0.3763	N.A	N.A	0.52	0.0432
OTU: s_*Hydrogenophaga bisanensis*	− 0.91	0.0001	− 0.91	0.0001	− 0.89	0.0001	− 0.70	0.0035
OTU: s_*Massilia aurea*	− 0.86	0.0001	− 0.85	0.0001	− 0.83	0.0001	− 0.72	0.0025
OTU: s_*Bifidobacterium animalis subsp*. *lactis*	− 0.85	0.0001	− 0.93	0.0001	− 0.60	0.0161	N.A	N.A
OTU: s_*Pseudomonas zhaodongensis*	− 0.91	0.0001	− 0.66	0.0063	− 0.64	0.0086	− 0.75	0.0013
OTU: s_*Pseudomonas zeshuii*	− 0.79	0.0004	− 0.40	0.1216	− 0.88	0.0001	− 0.65	0.0077
OTU: f_*Oxalobacteraceae*	− 0.88	0.0001	− 0.47	0.0686	− 0.77	0.0008	− 0.65	0.0077
OTU: s_*Massilia namucuonensis*	− 0.87	0.0001	− 0.85	0.0001	− 0.81	0.0002	− 0.61	0.0139
OTU: g_*Bradyrhizobium*	− 0.89	0.0001	− 0.91	0.0001	− 0.73	0.0018	− 0.67	0.0057

*M1*-*3* mock communities 1–3, *r*_s_ Spearman’s rho

#### Sequencing accuracy

**Fig. 4:**
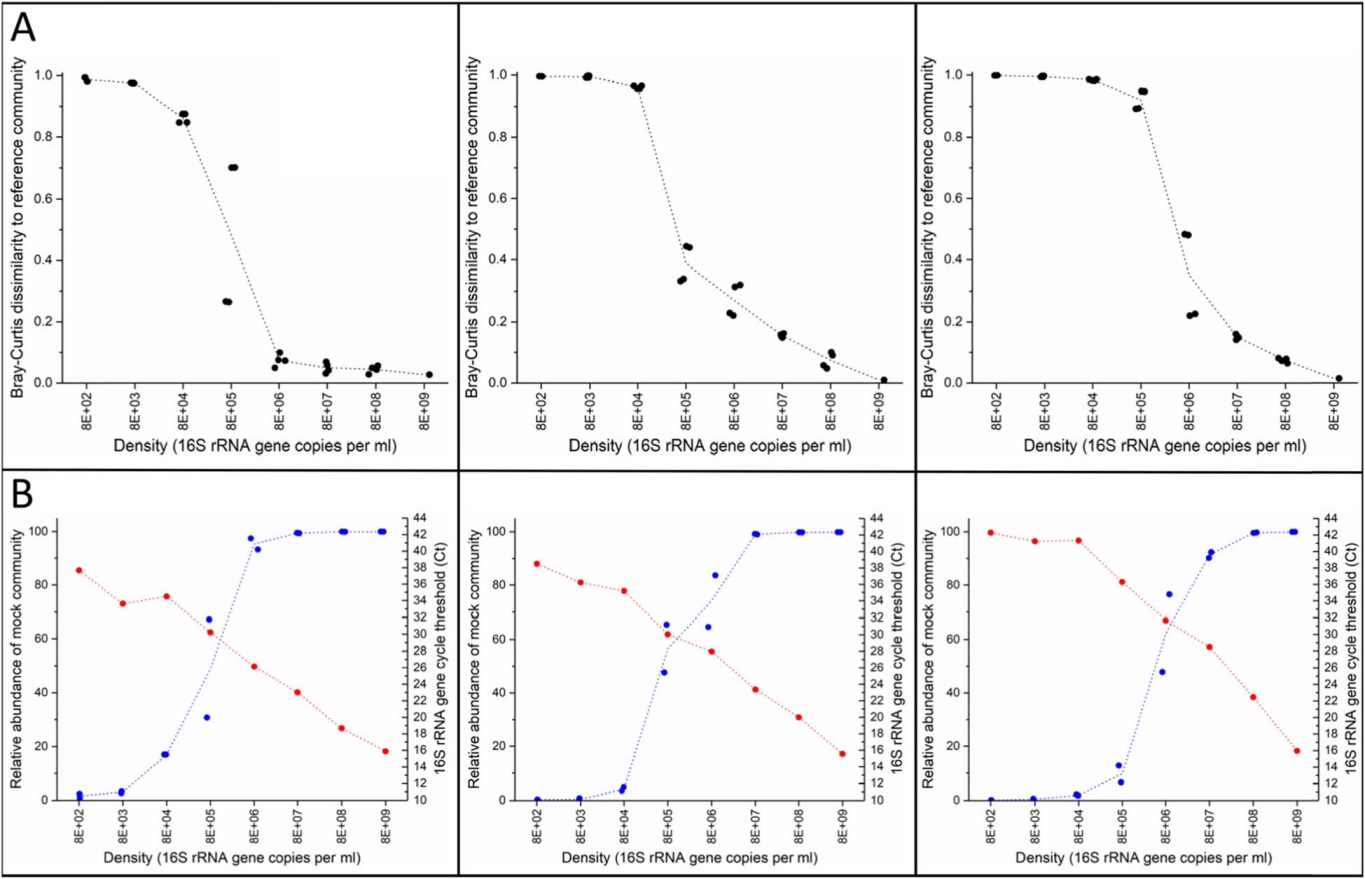
Sequencing accuracy across a range of densities. **a** Scatter plot showing the Bray-Curtis dissimilarity between the expected community composition and all input densities. Mock community 1 is shown on the left panel, mock community 2 is shown on the middle panel, and mock community 3 on the right panel. **b**. Plot of the relative abundance of members from the 3 mock communities (solid blue line) in comparison with the cycle threshold observed in the 16S qPCR (solid red line). ml = millilitre

We assessed sequencing accuracy by comparing taxonomic composition of a sample compared to the expected community composition (33/33/33% for community 1, 50/50% for community 2, and 55/24/15/5.5/0.5% for community 3) using Bray-Curtis dissimilarity (Fig. 4a). The mean degree of dissimilarity is low (BCI < 0.3) for input densities ≥ 8E+ 06 16S copies/ml for all mock communities. The mean dissimilarity is 0.59 at a density of 8E+ 05 16S copies/ml and ≥ 0.75 for densities less than 8E+ 05 16S copies/ml. This drop-off in accuracy occurs at the same density as a marked increase in the relative abundance of mock community taxa (Fig. 4b).

#### Sequencing precision

**Fig. 5:**
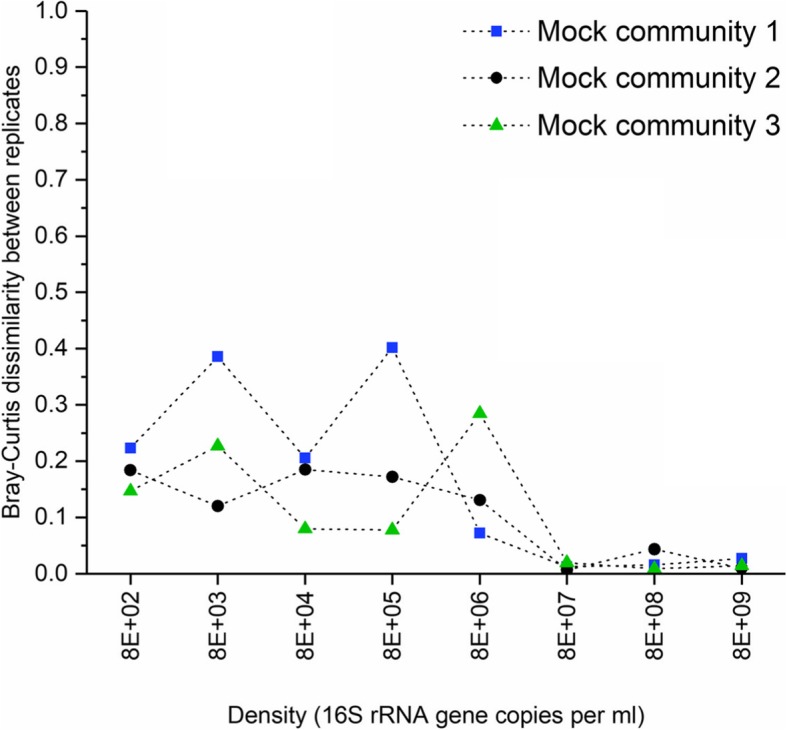
Sequencing precision across a range of densities. This plot shows the degree of dissimilarity between technical replicates, for all mock communities, measured using the Bray-Curtis dissimilarity index at each input density. ml = millilitre

We assessed sequencing precision by comparing taxonomic composition between technical replicates using Bray-Curtis dissimilarity (Fig. 5). In this case, technical replicates are DNA extractions performed in parallel, using the same biological sample as input for DNA extraction. Composition of technical replicates was similar (BCI < 0.25) for all mock communities at all input densities except at 8E+ 03 and 8E+ 05 16S copies/ml for the first mock community (BCI = 0.35 and 0.37, respectively) and 8E+ 06 16S copies/ml for the third mock (BCI = 0.28). At a density above 8E+ 07 16S copies/ml, the composition between replicates is highly concordant (BCI < 0.05), for the 3 mock communities.

### Effect of pre-sequencing sample concentration on sequencing accuracy and precision

**Fig. 6:**
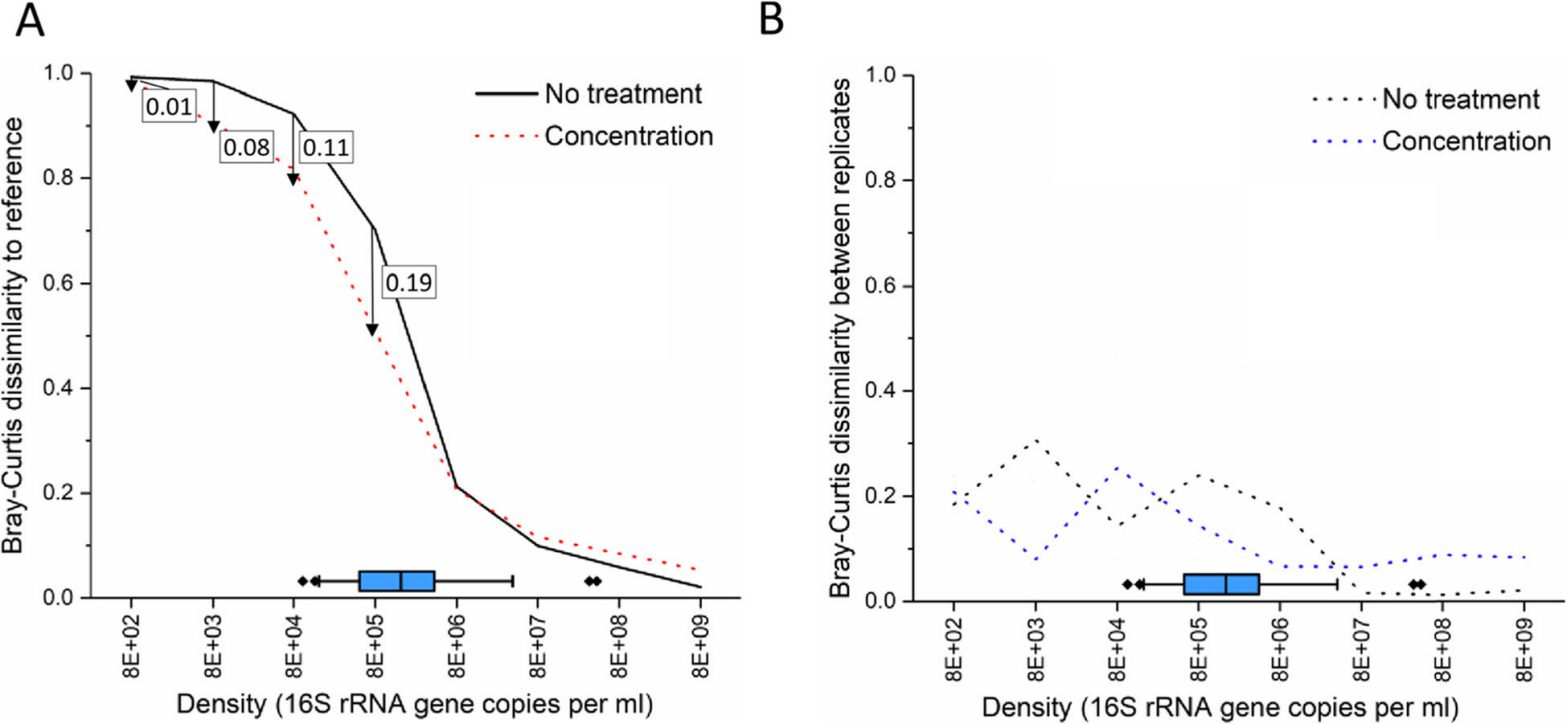
Treatment effect on Bray-Curtis dissimilarity between reference communities and all tested input densities. **a** Scatter plot showing the effect of pre-sequencing treatment on sequencing accuracy. The difference in dissimilarity to reference (ΔBCI) between each treatment is shown as a ruler for each dilution (if ΔBCI > 0). For reference, densities observed in our set of BALF samples are shown as a box chart at the bottom of the graph (the left and right of each box represent 75th and 25th percentiles, respectively; the left and right of each whisker represent 90th and 10th percentiles, respectively; line across inside of each box represents the median value, and the diamond beyond whiskers means outliers). **b**. Scatter plot showing the effect of pre-sequencing treatment on sequencing precision between replicates across a range of input densities

We tested the effect of concentration on the sequencing accuracy for mock communities 1 and 3 (Fig. 6**)**. Bronchoscopies are commonly performed with 50–100 ml of saline but recovery is highly variable and resulting samples often range between 1 and 50 ml of fluid. Most common DNA extraction methods, such as those recommended for the Human Microbiome Project, have an upper limit for sample input volume, and this is particularly limiting for samples presenting low bacterial load such as BALF. We tested the impact of physical concentration on sequencing accuracy on a range of densities prepared from mock communities 1 and 3. At sample densities between 8E+ 03 16S copies/ml and 8E+ 05 16S copies/ml, concentration alone improved taxonomic similarity to the expected distribution of the input sample (Wilcoxon signed rank test comparing BCI from untreated and concentrated samples; *P* < 0.05), but not at higher (> 8E+ 06 16S copies/ml) and the lowest (< 8E+ 03 16S copies/ml) input densities.

### Features of contaminating OTUs

Since our approach allows us to definitively label taxa as a mock community member or a contaminant, we were able to identify features specific to contaminants, with the goal of using them to subsequently filter tables and improve sequencing accuracy and precision. Contaminants had several features that distinguished them from mock community members.

#### Contaminant relative abundance is inversely correlated to input sample 16S density

**Fig. 7:**
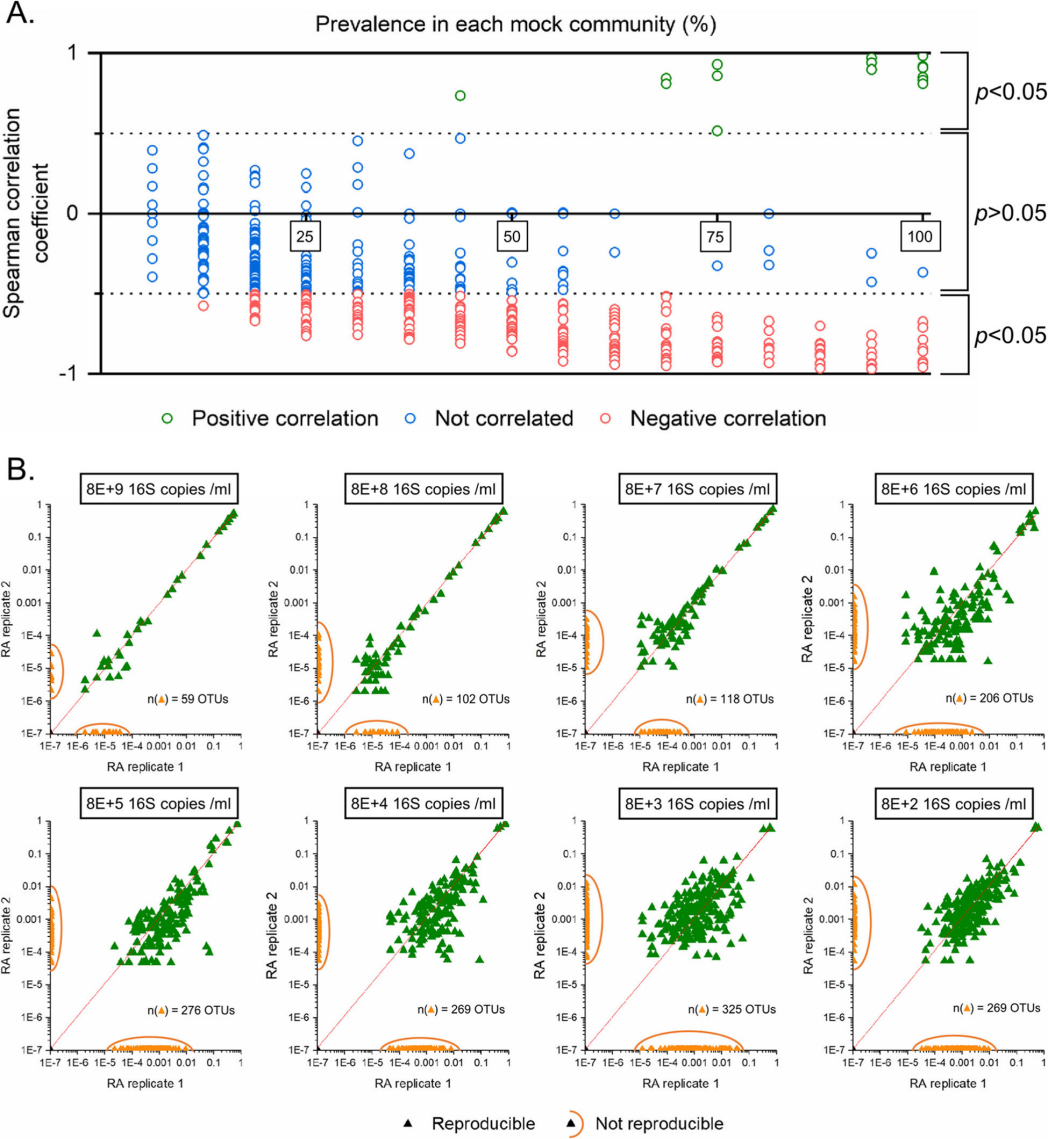
OTU features in mock communities. **a** Relative abundances of OTUs correlated with sample input density (Feature 1). Each dot indicates an OTU, coloured by correlation with sample densities. **b** Scatter plot showing irreproducible OTUs across a range of sample densities (Feature 2). OTUs which are consistent between replicates are coloured in green, non-reproducible OTUs in orange

One hundred and thirty-four out of 159 contaminant OTUs with a prevalence in the dataset above 50% were negatively correlated with sample input density (Fig. 7a). At prevalence below 50%, 103 out of 736 contaminant OTUs were negatively correlated with density. We refer to this feature of contaminating taxa as ‘Feature 1’.

#### Contaminants have low reproducibility in technical replicates

The number of non-reproducible OTUs across the range of densities increases with decreasing sample density (Fig. 7b). The number of non-reproducible contaminants ranges from 59 to 325 OTUs across mock communities. We refer this feature of contaminating taxa as ‘Feature 2’.

### Contribution of OTUs with Features 1 and 2 to mock communities across input sample densities and the impact of their removal on precision and accuracy

**Fig. 8:**
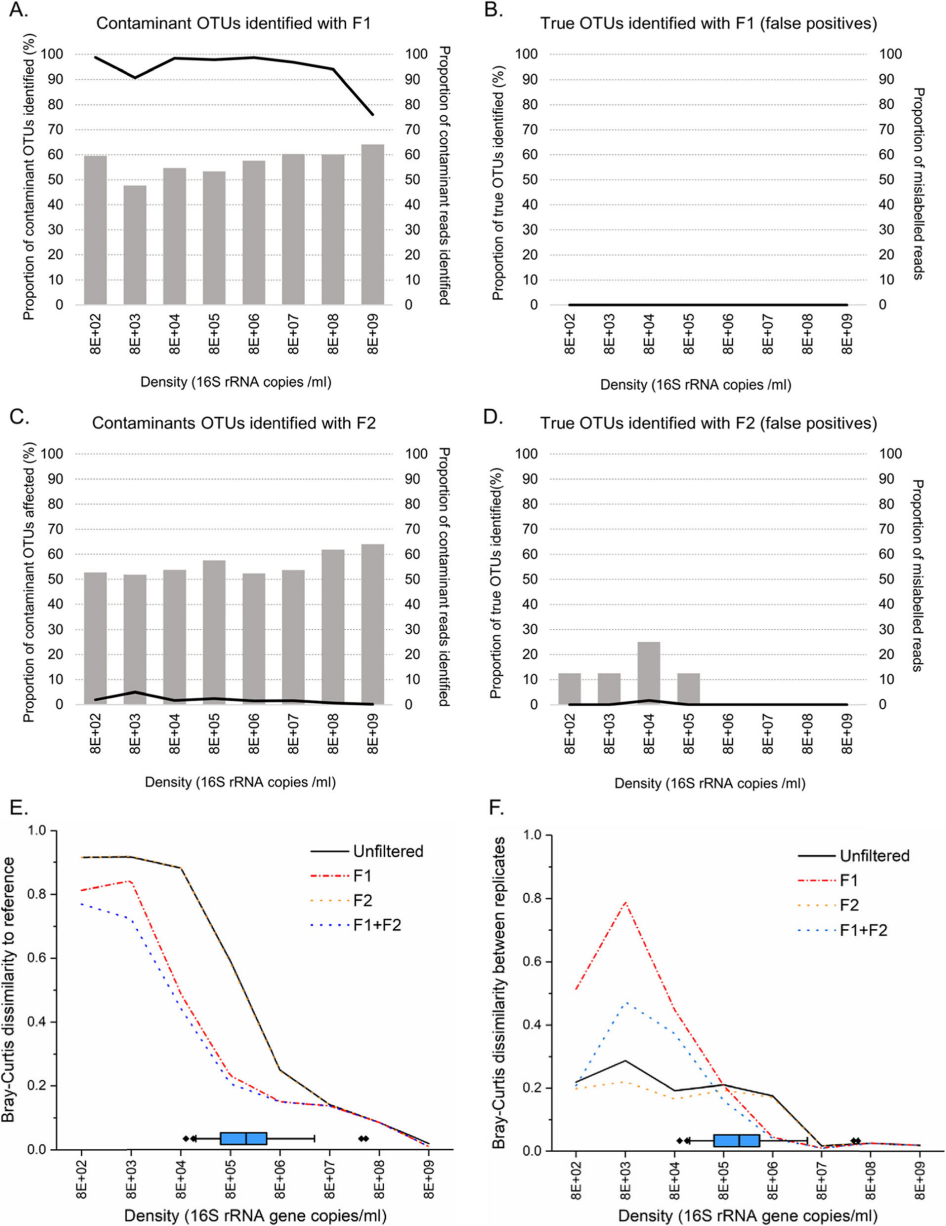
Quantitative effects of removing OTUs with features of contaminants on mock community samples. **a**–**d**. Bar chart showing the proportion of OTUs that are negatively correlated with density (Feature 1, F1) or that were not reproducible between technical replicates (Feature 2, F2), along with the proportion they represent within their target groups (contaminant or true taxa). The black line indicates the cumulative amount within each target group (contaminants or true taxa) corresponding to the OTUs identified with each filtering approach. **e** Comparison of post-filtering effect on sequencing accuracy across a range of densities. **f** Sequencing precision across range of densities before and after removal by feature

Between 47 and 64% of contaminants demonstrated Feature 1, accounting for 76–99% of the cumulative relative abundance of all contaminating OTUs (Fig. 8a). No mock community taxa demonstrated this feature (Fig. 8b). Between 51 and 64% of contaminating OTUs demonstrated Feature 2, accounting for between 0.1 and 6.5% of the cumulative relative abundance of all contaminating OTUs (Fig. 8c). Two mock community members (*Streptococcus* and *Gemella*) demonstrated Feature 2, but only in low abundance samples (< 8E+ 05 16S gene copies/ml, Fig. 8d).

Removal of OTUs with Feature 1 improved accuracy in samples with a density below 8E+ 07 16S copies/ml (Fig. 8e) but worsened precision for samples at lower input densities (< 8E+ 05 16S copies/ml, Fig. 8f). Removal of OTUs with Feature 2 had no impact on accuracy but retained or slightly improved precision at low densities. Combining the filters improved accuracy with a smaller trade-off in precision at lower density than removal of taxa with Feature 1 only (Fig. 8f). Importantly, sequencing accuracy at a sample density below 8E+ 03 16S copies/ml remained low (BCI > 0.5) after treatment and filtering, indicating the data obtained from clinical samples presenting similar bacterial densities should be interpreted with caution.

#### Features of taxa that are both common contaminants and known colonizers of the human airway

**Fig. 9:**
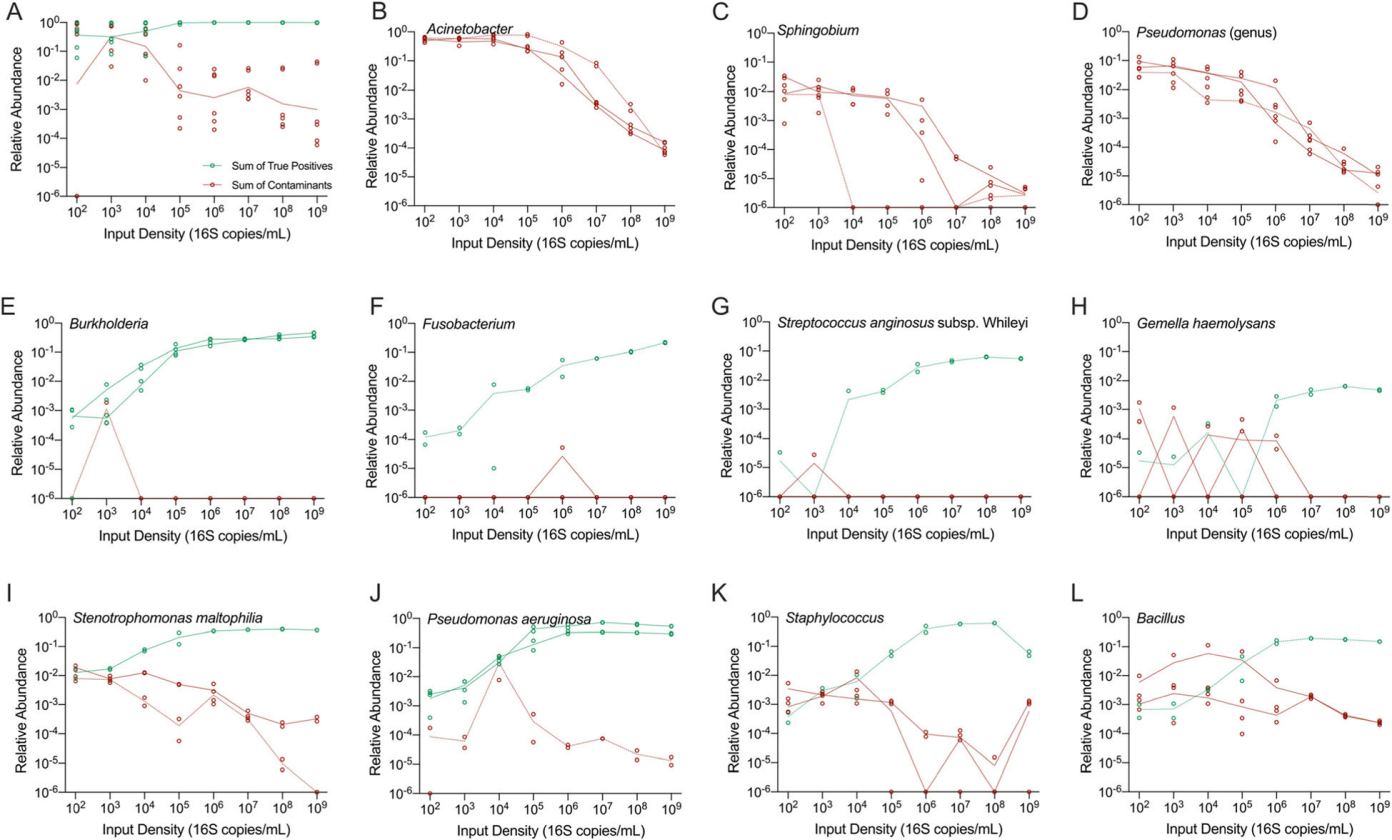
Status of mock species and two known contaminants in each community. **a** Scatter plot showing the sum of true positives against contaminants across a range of densities. **b**–**d** Plot showing the status of two true contaminants in each mock community. **e**–**l** Plot showing the status of each true taxon in the three mock communities

Multiple bacterial taxa such as *Pseudomonas*, *Acinetobacter*, and *Stenotrophomonas* are both common lung pathogens/colonizers and frequent reagent contaminants. Since no single mock community taxon was present in all three input communities, we were able to assign mock community OTUs contaminant or true-positive status in each sample and assess their relative abundance across the full input density range (Fig. 9). The likelihood that an OTU represented a true positive varied by OTU and by input density. *Burkholderia*, *Fusobacterium*, and *Streptococcus anginosus* were consistently true positives across the full density range. True-positive *Pseudomonas aeruginosa* was generally distinguishable from the distinct contaminant *Pseudomonas* with our sequencing methods, and, if identified at the species level, was likely to be a true positive at a relative abundance of > 0.001. Several other taxa, however, including *Gemella*, *Stenotrophomonas maltophilia*, *Bacillus*, and *Staphylococcus* were reliably true positives only at higher relative abundance (> 0.001) and only in higher density samples (> 8E+ 04 16S copies/ml), indicating that relative abundance and input sample density must both be incorporated into interpretation of these taxa in BALF and that lack of control for input sample density may lead to a biased interpretation of relative abundance of these taxa.

### Features of OTUs across density ranges in BALF samples

 We next analysed 50 post-transplant BALF samples obtained from surveillance bronchoscopies to identify whether OTUs in BALF in this population reflect the features of contaminants observed in our mock communities. Using 50 BALF samples, we assessed the correlation between relative abundance of each OTU and samples input density to identify contaminants.

**Fig. 10:**
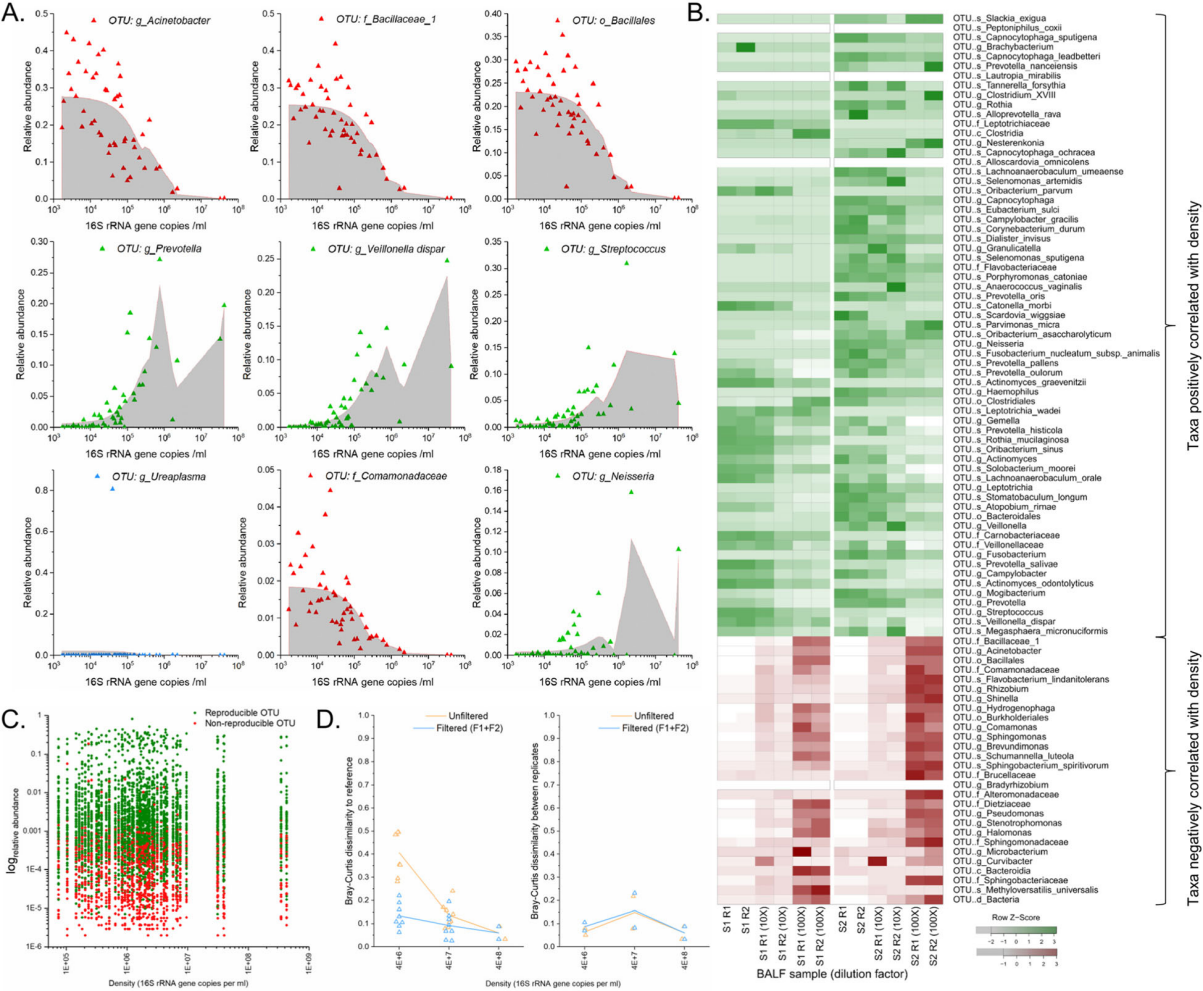
Contaminant features in a set of BALF samples. **a** Taxa representing more than 1% of the total set of BALF samples, coloured by whether they are negatively correlated (red), positively correlated (green), or not correlated (blue) with input sample bacterial density. The shaded area represents the locally weighted scatterplot smoothing (LOWESS). **b**. The heatmap of relative abundance z-score for OTUs which were correlated with samples input density. Positively correlated OTUs (putative true positives) are represented with a gradient of green while negatively correlated OTUs (suspected contaminants) are represented with a gradient of red. **c** Reproducibility of OTUs in BALF samples plotted by sample density and log relative abundance. **d** Effect of post-sequencing filtering on sequencing accuracy and precision of a dilution series of a single BALF sample, comparing dilutions to the composition of the highest density sample

The relative abundance across input densities of OTUs representing at least 1% of the overall sequence abundance are shown in Fig. 10a. Four OTUs (*Acinetobacter*, *Bacillaceae*, *Bacillales*, and *Commamonadaceae*) were negatively correlated with density, consistent with contaminant Feature 1, while taxa known to colonize the airways (*Prevotella*, *Veillonella dispar*, *Streptococcus*, and *Neisseria*) were positively correlated with density. Interestingly, *Ureaplasma* was detected at high relative abundance in a single sample but lacked either feature of contaminants. All OTUs with positive or negative correlation with sample density are shown in Fig. 10b. Negatively correlated OTUs largely represent common contaminants in low biomass samples, whereas positively correlated taxa represent known colonizers of the human airway.

We then assessed which OTUs presented the second feature of contaminants across the set of BALF samples (Fig. 10c). Above a relative abundance of 1E− 3, (the threshold above which OTUs were more reliably true positive taxa in our mock communities), 89% of the OTUs were reproducible between technical replicates, but below this threshold, the majority of OTUs were not reproducible (Additional file 1).

Using dilution series of two BALF samples presenting a density of ~ 4E+ 08 16S copies/ml, we assessed the effect of the removal of taxa with contaminant Features 1 or 2, or either feature combined filters on sequencing accuracy and precision (Fig. 10d). Sequencing accuracy is significantly improved at 4E+ 06 16S copies/ml with a BCI dissimilarity to reference decreasing to 0.13, instead of 0.41 for the unfiltered sample (Mann-Whitney *U* test; *P* < 0.01). At 4E+ 07 16S copies/ml, accuracy is slightly improved with a BCI of 0.09 for the filtered samples instead of 0.14 for the unfiltered samples. At the highest density (4E+ 08 16S copies/ml), filtered samples are similar to the unfiltered references. The dissimilarity between replicates remained similar across tested dilutions between filtered and unfiltered replicates.

## Discussion

Unlike stool or other samples with consistently high bacterial loads, BALF have input bacterial densities that range over several orders of magnitude and include very low densities. The analysis of the microbiome present in low-density samples is particularly challenging due to the ubiquitous presence of contaminants [18, 19, 24]. Using three different mock communities over a density range representative of BALF across numerous health/disease states, we ascertained the features of contaminating OTUs across a representative range of input sample densities, determined density-specific accuracy and precision, and determined the effect of simple pre-sequencing and contaminant removal strategies on sequencing accuracy and precision. We then confirmed the observations from our mock communities using BALF samples.

We found strong sample density-dependence of sequencing accuracy and precision. At a density below ~ 8E+ 06 16S copies/ml in unfiltered samples, the observed signal was derived from both bacterial taxa from the mock communities and contaminants. We identified features that distinguished contaminants from true positive taxa, including that they were negatively correlated with input density and that they showed low inter-replicate reproducibility. These features facilitated identification of a large proportion of contaminating OTUs and reads but could not be used to distinguish between taxa that are both common contaminants and airway pathogens/colonizers, indicating that current, commonly used amplicon sequencing methods will be limited for the quantitation of these taxa, especially in low-relative abundance or in low-density samples. In spite of these limitations, however, identification and removal of reads from these OTUs based on these features can improve accuracy without a significant impact on precision for samples with an input density above ~ 8E+ 04 16S copies/ml. It is important to note that with relative abundance compositional data, samples that are dissimilar based on density and relative abundance of contaminants may appear compositionally similar if reads from contaminants are removed. While this form of filtering may improve accuracy and precision, it potentially obscures differences between samples that may be informative, particularly those due to differences in the abundance of ‘true positive’ taxa that are lower in relative abundance in only one group of interest. This is an inherent limitation of relative abundance data but is an important consideration in the interpretation of samples of variable density and may need to be addressed with complementary methods (such as absolute quantitation of taxa or quantitative normalization).

Our study has several important limitations. Not all known colonizers of the human airway nor all community composition types (e.g. taxon number or distribution) were represented in our mock communities. Our experiments represent only a single centre, and contaminants may vary by site and protocol-specific factors. Importantly, our method will not distinguish between contaminants introduced at the time of sample collection and those introduced during sample handling and processing.

## Conclusions

Based on our observations, the following factors should be considered when analysing BALF, especially when bacterial density is low in some samples:

1. We recommend pre-screening of sample bacterial densities to predict expected sequencing accuracy and precision for any given sample set.
2. For samples with densities comprised between 8E+ 04 and 8E+ 06 16S copies/ml BALF, we recommend sample concentration as well as the use of sequencing replicates and dilution series.
3. Identification of OTUs inversely correlated with density or with poor technical replicability is a useful strategy to improve sequencing accuracy and precision. However, removal of reads should be considered within the context of analytical goals and the limitations associated with the use of relative abundance data. We suggest that removal of OTUs with features of contaminants be combined with other analytical approaches such as absolute quantitation of key taxa (e.g. by qPCR), quantitative normalization (e.g. with 16S qPCR), or comparisons of relative abundance data of only ‘true-positive’ taxa without removal of putative contaminants.
4. Given the high precision between biological replicates for samples above 8E+ 06 16S copies/ml, sequencing replicates might not be necessary for all samples, and the sequencing strategy and costs can be optimized depending on input bacterial density.
5. Finally, we encourage each laboratory to identify the specific performance characteristics of their own experimental environment and methods, using dilution series of samples with known composition or high input density, covering the entire range of sample densities in their sample set.

## Methods

### Density of BALF samples

To calibrate the range of densities of our mock communities, we retrieved a set of 51 selected post-transplantation BALF samples obtained from bronchoscopies from the Toronto Lung Transplant Program (TLTP) biobank, reasoning that this population is both highly sampled and has a diversity of both infectious and non-infectious complications of transplantation. Raw, unspun, and unfiltered, BALF samples were used for analyses. Our programme’s collection protocol was published previously [25].

### Mock community

Bacterial isolates were obtained from the American Type Culture Collection (ATCC, Manassas, VA, USA). Glycerol stocks were prepared upon arrival as recommended by ATCC. *Pseudomonas aeruginosa* str. PAO1, *Burkholderia multivorans* (ATCC 17616), *Stenotrophomonas maltophila* (ATCC 13637), *Staphylococcus aureus* (ATCC 12600), *Fusobacterium nucleatum* (ATCC 23726), *Streptococcus anginosus* (ATCC 33397), *Bacillus halodurans* (ATCC BAA-125), and *Gemella haemolysans* (ATCC 10379) were grown (aerobically or anaerobically) overnight in Tryptic-Soy broth (TSB) at 37 °C and subsequently quantified on Tryptic-Soy agar plates. Culture broths were pooled to a density of ~ 8E+ 09 16S copies/ml. A series of seven tenfold dilutions was prepared resulting in sample densities ranging from 8E+ 09 to 8E+ 02 16S copies/ml. Three mock communities were prepared, the first being composed of an equimolar ratio of *P*. *aeruginosa*, *B*. *multivorans*, and *S*. *maltophilia*. The second community was composed of *P*. *aeruginosa* and *B*. *multivorans*, each accounting for 50% of the community composition. The third community was composed of *S*. *aureus* (55%), *F*. *nucleatum* (24%), *B*. *halodurans* (15%), *S*. *anginosus* (5.5%), and *G*. *haemolysans* (0.5%).

### Sequencing controls

Four types of negative controls were used in this study. The first was an aliquot of the elution buffer (H_2_O) used in the extraction protocol (NTC1-2). The second control was an aliquot of TSB medium used to cultivate the mock species (NTC3-4). The third was elution buffer (H_2_O) which was extracted along other samples (NTC5-6). The final control was H_2_O which was concentrated and subsequently extracted (NTC7-8). All controls were sequenced in duplicate. All species included in the mock communities were also sequenced individually, and the resulting OTUs were used to differentiate mock species from contaminating taxa.

### DNA isolation and quantification

Nucleic acids were isolated from 250 μl of sample using a PowerSoil DNA isolation kit (MO-BIO; Carlsbad, CA, USA) following the manufacturer’s instructions except for the elution step which was done in 60 μl purified water. Densities were measured using a 16S quantitative polymerase chain reaction (qPCR [26];) and a standard (*Pseudomonas aeruginosa* str. PAO1), and the number of 16S copies/ml was inferred using the URI Genomics & Sequencing Center online calculator (http://cels.uri.edu/gsc/cndna.html). 16S qPCR primers and conditions are described in Additional file 2. qPCR reactions were carried out in a volume of 11 μl using the TaqMan Gene Expression Master Mix (Applied Biosystems, Foster City, CA, USA) according to the manufacturer’s protocol.

### Concentration

Samples were concentrated using Amicon Ultra-15 Centrifugal Filter Units with the 30-kDa filter (MilliporeSigma, Darmstadt, Germany). A tenfold concentration factor was obtained by concentrating 5 ml of sample into a volume of 500 μl.

### 16S rRNA gene sequencing

The V4 hypervariable region of the 16S rRNA gene was amplified using a universal forward sequencing primer and a uniquely barcoded reverse sequencing primer to allow for multiplexing [27]. Amplification reactions were performed using 12.5 μl of KAPA2G Robust HotStart ReadyMix (KAPA Biosystems), 1.5 μl of 10 μm forward and reverse primers, 8 μl of sterile water, and 1.5 μl of DNA. The V4 region was amplified by cycling the reaction at 95 °C for 3 min, 30× cycles of 95 °C for 15 s, 50 °C for 15 s, and 72 °C for 15 s, followed by a 5-min 72 °C extension. All amplification reactions were done in triplicate, checked on a 1% agarose TBE gel, and then pooled to reduce amplification bias. Pooled triplicates were quantified using Quant-it PicoGreen dsDNA Assay (Thermo Fisher Scientific) and combined by even concentrations. The final library was purified using Ampure XP beads (Agencourt), selecting for the bacterial V4 amplified band. The purified library was quantified using Qubit dsDNA Assay (Thermo Fisher Scientific) and loaded on to the Illumina MiSeq for sequencing, according to manufacturer instructions (Illumina, San Diego, CA, USA). Sequencing was performed using the V2 (150 bp × 2) chemistry. Sequencing depths are reported in Additional file 3.

### Analysis of the bacterial microbiome

The UNOISE pipeline, available through USEARCH version 10.0.240, was used for sequence analysis [28–30]. The last base, typically error-prone, was removed from all the sequences. Sequences were assembled and quality trimmed using –fastq_mergepairs and –fastq_filter, with a –fastq_maxee set at 1.0 and 0.5, respectively. Assembled sequences less than 233 bp were removed. Following the UNOISE pipeline, unique sequences were identified from the merged pairs and sorted. Sequences were denoised and chimaeras were removed using the unoise3 command in USEARCH. Assembled sequences were then mapped back to the chimaera-free denoised sequences at 97% identity OTUs using the –usearch_global command. Taxonomy assignment was executed using SINTAX [31], available through USEARCH, and the SINTAX-compatible Ribosomal Database Project (RDP) database version 16, with the default minimum confidence cut-off of 0.8 [32]. OTU sequences were aligned using PyNast accessed through QIIME [33]. Sequences that did not align were removed from the dataset and a phylogenetic tree of the filtered aligned sequence data was made using FastTree [34].

### Removal of OTUs with features of contaminants as a filtering strategy for BALF samples

We applied three different filtering strategies based on features observed in contaminating OTUs. This included (1) the identification and removal of contaminating taxa based on the negative correlation between their relative abundances and sample input densities (Feature 1), (2) the identification and removal of singletons in technical replicates (Feature 2), or (3) the combined removal of OTUs with either of these features. To apply Filter F1, we converted raw absolute abundance tables to relative abundances. We subsequently measured the Spearman correlation between relative abundances and sample input density and tested for significance for both the mock sample set and the BALF set, independently. OTUs presenting significant negative correlation were labelled as contaminants. For filter F2, we assessed whether each bacterial taxon was present in both technical replicates and subsequently removed those which were found to be singletons. For filter F3, we first applied F1, followed by F2.

### Statistical analysis

Bray-Curtis dissimilarity indices were calculated using the ‘dissimilarity’ function from the Vegan R package version 2.5-2 [35]. Wilcoxon signed rank tests and Spearman correlations were calculated using XLSTAT 2019 (Addinsoft: Paris, France). Plots were generated using OriginPro 2017 (Northampton, MA, USA) and the R packages ggplot2 version 3.0.0 [36] and reshape2 version 1.4.3 [37].

## Supplementary information

**Additional file 1:** Reproducibility of OTUs across duplicates.

**Additional file 2:** 16S qPCR primers and conditions.

**Additional file 3:** Study IDs and metadata.

## Data Availability

Sequence data that support the findings of this study have been deposited in the NCBI Short Read Archive with the primary accession code PRJNA505523.
